# Supplementary material for: Glassy thermal conductivity in Cs3Bi2I6Cl3 single crystal
Source: Nat Commun. 2022 Aug 27;13:5053. doi: 10.1038/s41467-022-32773-4 (PMC9420152; doi:10.1038/s41467-022-32773-4)
Supplement: Supplementary file 1 — Supplementary Information [file 41467_2022_32773_MOESM1_ESM.pdf]

Supplementary Information

Glassy Thermal Conductivity in $Cs_3Bi_2I_6Cl_3$ Single Crystal

Paribesh Acharyya,[1] Tanmoy Ghosh,[1] Koushik Pal,[2,3] Kewal Singh Rana,[4] Moinak Dutta,[1] Diptikanta Swain,[5] Martin Etter,[6] Ajay Soni,[4] Umesh V. Waghmare,[2,7] and Kanishka Biswas[1,7,]*

[1]New Chemistry Unit and [2]Theoretical Science Unit, Jawaharlal Nehru Centre for Advanced Scientific Research (JNCASR), Jakkur P.O., Bangalore 560064, India

[3]Present address: Department of Materials Science and Engineering, Northwestern University, Evanston, Illinois 60208, United States

[4]School of Basic Sciences, Indian Institute of Technology Mandi, Mandi, Himachal Pradesh 175075, India

[5]Institute of Chemical Technology-IndianOil Odisha Campus, Bhubaneswar 751013, India.

[6]Deutsches Elektronen-Synchrotron (DESY), 22607 Hamburg, Germany

[7]School of Advanced Materials and International Centre for Materials Science, Jawaharlal Nehru Centre for Advanced Scientific Research (JNCASR), Jakkur P.O., Bangalore 560064, India

*Corresponding author: kanishka@jncasr.ac.in

Detailed Materials and Methods

Materials. Cesium (I) chloride (CsCl, 99.9%, Sigma Aldrich) and bismuth (III) iodide (BiI_3, 99.998%, Sigma Aldrich) were used for synthesis without further purification.

Bridgman crystal growth. Single crystal of $Cs_3Bi_2I_6Cl_3$ was grown using a Bridgman furnace. In order to grow a large single crystal, stoichiometric amount of CsCl (2.0989 g) and BiI_3 (4.9011 g) were sealed in a 10 mm conical quartz tube under high vacuum (10^{-6} Torr). The ampule was kept at 750 ^{o}C for 48 hrs and the ampule was then moved through a temperature gradient from 600 ^{o}C to 480 ^{o}C at a speed of 1 mm/hr. Finally, the sample was slowly cooled to room temperature in 120 hrs.

Single crystal X-ray diffraction (SCXRD). Data were collected at room temperature (298 K) using a Bruker D8 VENTURE diffractometer equipped with a PHOTON detector and graphite-monochromatic Mo-Kα radiation (λ = 0.71073 Å, 50 kV, 1 mA). APEX III software was used to collect, reduce, and integrate the raw data. The structure solution was obtained from SHELXS and refined by using SHELXL included in the WinGX suite.[1-3] The crystallographic details are given in Table S1 and S2.

Powder X-ray diffraction (PXRD). Room temperature XRD patterns of $Cs_3Bi_2I_6Cl_3$ for two different directions (parallel and perpendicular to the Bridgman growth direction) were collected on a Bruker D8 diffractometer using Cu Kα radiation (λ= 1.5406 Å). Synchrotron powder X-ray diffraction data was collected from finely ground powder using a synchrotron X-ray beam of λ = 0.7762 Å, at BL-18B (Indian beamline), Photon Factory, KEK, Tsukuba, Japan. A Si (111) double crystal monochromator was used to set the energy of the beam and analyzed with standard Si (640b NIST). The measurement was performed in Bragg−Brentano geometry with an anti-scattering slit (350 μm), a divergence slit (300 μm), and a receiving slit (300 μm). Refinement of synchrotron powder X-ray diffraction data (Supplementary Fig. 2a) was performed using the FullProf program.[4]

Thermal conductivity. First, large single crystal was cut in perpendicular and parallel to Bridgman growth direction (see Supplementary Fig. 1) by a diamond wire saw (Diamond wire saw 3500 premium) to measure the thermal conductivity. Thermal conductivity measurement was

carried out in the temperature range of 2-400 K. The low temperature thermal conductivity (2-300 K) was measured for a rectangular bar shaped specimen with dimensions of ~2 x 2 x 8 mm^3 using a physical properties measurement system (DynaCool PPMS, Quantum Design). Above room temperature (300-400 K), thermal diffusivity, D, was measured by laser flash diffusivity technique using a Netzsch LFA-457 instrument (Supplementary Fig. 3). Typical square (~8 x 8 mm^2) and coin (~8 mm diameter) shaped samples of thickness less than 2 mm were used for the measurement of thermal transport properties above room temperature and thermal conductivity (κ) was estimated using the relation, $\kappa = D \times C_p \times \rho$, where ρ is the density of the sample and C_p is the specific heat. The obtained density of the single crystal is ~98 % of the theoretical density. We have used the Dulong–Petit C_p (0.21 J/g/K) for the estimation of thermal conductivity in the 300-400 K range. The error in thermal conductivity measurement was determined using the standard deviation in thermal conductivity as per the standard protocol used in Quantum Design PPMS. The measurement error for LFA thermal conductivity is 5%.

Sound velocity measurement. The longitudinal (v_l) and transverse (v_t) sound velocities were measured using disc-shaped and square-shaped samples, respectively, with an Epoch 650 Ultrasonic Flaw Detector (Olympus) instrument with the transducer frequency of 5 MHz. The minimum lattice thermal conductivity (κ_{min}) was then calculated using the Cahill's Model [5]:

$$\kappa_{min} = 1.21\mathrm{n}^{2/3} k_B \frac{1}{3}(v_l + 2v_t) \ldots\ldots\ldots\ldots\ldots\ldots..(1)$$

We have calculated the diffuson mediated thermal conductivity (κ_{diff}) using the formula below [6]:

$$\kappa_{diff} = 0.76\mathrm{n}^{2/3} k_B \frac{1}{3}(v_l + 2v_t) \ldots\ldots\ldots\ldots\ldots\ldots..(2)$$

where k_B is the Boltzmann constant and n is the number density of atoms.

Poisson ratio (ν_p) and the Grüneisen parameter (γ) were calculated using equations below (see Table S3) [7]:

$$\nu_p = \frac{1-2(v_t/v_l)^2}{2-2(v_t/v_l)^2} \ldots\ldots\ldots\ldots\ldots..(3)$$

$$\gamma = \frac{3}{2} \cdot \frac{1 + \nu_p}{2 - 3\nu_p} \dots \dots \dots \dots \dots \dots \dots . \dots . (4)$$

Further, the average sound velocity (v_a) was also estimated from the Debye temperature (Θ_D) using the following equation:[8]

$$\Theta_D = \frac{h}{k_B} \left(\frac{3N}{4\pi V_u} \right)^{1/3} v_a \dots . (5)$$

where h is Planck's constant, N is the number of atoms in a unit cell, V_u is the unit-cell volume. The estimated average sound velocity (v_a) was determined to be ~ 1200 m/s which is close to the experimental value (Table S3).

We have determined the bulk (B) and shear (G) moduli using longitudinal (v_l) and transverse (v_t) sound velocities applying the equation below,[9] and compared them to theoretically estimated elastic moduli as shown in Table S4.

$$G = {v_t}^2 \rho \dots . (6)$$

$$B = {v_l}^2 \rho - \frac{4}{3} {v_t}^2 \rho \dots . (7)$$

Heat capacity measurement. Low-temperature heat capacity (C_p) of $Cs_3Bi_2I_6Cl_3$ was measured in a Physical Property Measurement System (PPMS, DynaCool) in the temperature range of 2-50 K. We have used Debye-Einstein model to fit our data. The combined Debye-Einstein model can be expressed as:[10,11]

$$\frac{C_p}{T} = \gamma + \beta T^2 + \sum_n \left(A_n \left(\theta_{E_n}\right)^2 . (T^2)^{-3/2} . \frac{e^{\theta_{E_n}/T}}{\left(e^{\theta_{E_n}/T} - 1\right)^2} \right) \dots \dots . (8)$$

The coefficient of the first term on the right-hand side in the equation is the Sommerfeld coefficient γ which represents the electronic contribution and the coefficient for the second term, β, depicts the lattice contribution. The third term in the equation represents contributions from the Einstein oscillators corresponding to the localized lattice vibrations where the A_n is the prefactor and θ_{E_n} is the Einstein temperature of the nth Einstein oscillator mode.

The Debye temperature is estimated using the relation $\beta = C(12\pi^4 N_A k_B/5) \cdot (\Theta_D)^{-3}$ where C can be defined as , $C = 1 - \sum_n A_n/3NR$, (N = number of atoms/ formula unit and R = 8.314 J $mol^{-1}K^{-1}$, universal gas constant) and N_A, and k_B are the Avogadro's number, and Boltzmann constant respectively.

Raman spectroscopy. Horiba Jobin-Yvon LabRAM HR evolution Raman spectrometer was used for Raman measurements in back scattering geometry with excitation laser 785 nm (1.58 eV) and 633 nm (1.96 eV), large working distance 50 x objective, grating 1800 lines/mm and Peltier cooled CCD detector. The ultra low-frequency filters were used to detect the low-frequency modes (below 50 cm^{-1}) for 633 nm excitation wavelength. The temperature dependent (4-300 K) Raman study was conducted by using Montana cryostat through 785 nm laser and power ~0.80 mW. All the observed spectra were baseline corrected, normalized with respect to the intense peak (59 cm^{-1}) and fitted with the Lorentzian function.

Field emission scanning electron microscopy (FESEM) in back-scattered electron (BSE). FESEM-BSE images were taken using ZEISS Gemini SEM – Field Emission Scanning Electron Microscope. Back-scattered electron (BSE) imaging in field emission scanning electron microscope (FESEM), energy dispersive X-ray (EDX) spectra and elemental color mapping with EDX (Supplementary Fig. 2b-e) show the homogeneous nature of the sample with uniform distribution of the compositional elements (Cs, Bi, I and Cl).

Thermogravimetric analysis (TGA). TGA experiments were carried out using a 2 STAR TGA instrument. Powdered samples were heated in the temperature range of 300-1247 K in N_2 atmosphere at a rate of 5 K/min. Thermal gravimetric analysis (TGA) further ensures that the compound is thermally stable up to 673 K, after which it starts to decompose due to sublimation of BiI_3 (melting point of BiI_3 is 681 K) as depicted in Supplementary Fig. 2f.

Optical spectroscopy. PerkinElmer, Lambda-900 UV/Vis/near-IR spectrometer was used to measure the optical bandgap in reflectance mode within the range of 250-800 nm. The absorption data (α/S) of $Cs_3Bi_2I_6Cl_3$ was estimated from reflectance data using Kubelka–Munk equation; $\alpha/S = (1-R)^2/(2R)$; where R, α, and S denote reflectance, absorption and scattering coefficients, respectively. Optical absorption study reveals an insulating nature of $Cs_3Bi_2I_6Cl_3$ with the absorption edge at ~2.0 eV (Supplementary Fig. 4).

Synchrotron X-ray Pair Distribution Function (X-PDF). Samples were finely ground with an agate mortar pestle and then filled in capillary of 0.6 mm diameter for performing synchrotron X-ray PDF measurements. Both ends of capillaries were sealed using an adhesive. A Perkin Elmer XRD1621 area detector was used to record the diffraction data. To subtract the background, data sets of empty capillaries were collected. A dark measurement prior to each data collection was done and Lanthanum Hexaboride (LaB_6) was taken as a standard for calibration. The wavelength of the beam was fixed at 0.20742 Å. The data was taken at the P02.1 beamline of PETRA III, DESY, Germany.[12]

G(r) defines the probability of finding nearest neighbor bonding at a certain distance *r* in the material, G(r) was obtained via Fourier transformation of the scattering structure function, F(Q) [13]

$$G(r) = \frac{2}{\pi}\int_{\mathrm{Qmin}}^{\infty} \mathrm{F(Q)\ sinQr\ dQ} \quad \ldots.(9)$$

where Q represents the momentum transfer of the scattering particle. F(Q) is obtained from the scattering data and is related to the structure function S(Q) as F(Q) = Q[S(Q)-1]. Initial data reduction was done using DAWN[14] and PDFgetX3[15] software. The processing parameters used for the data conversion are: Q_{min} = 0.0 Å $^{-1}$, Q_{max} = 21.5 Å $^{-1}$, r_{min} = 1.0 Å and r_{max} = 30 Å.

Simulation of the experimental PDF data was done using the PDFgui[16] software. The data at 300 K was initially modelled using a *P*-3*m*1 model. The refinement parameters were the scale, linear atomic correlation factor, lattice parameters, and the thermal displacement values. For positional refinements of each atom, the allowed positions were refined along with the aforementioned parameters. The *r* value of the experimental data was taken from 2 – 30 Å for the simulation. The first peak of the G(r) *vs. r* plot represents the nearest atom-atom correlation, the second peak corresponds to second nearest atom-atom distances and so on.

The thermal parameters of the temperature dependent PDF data are refined such a way that they show same values along the a and b-axis for all the atoms. The other temperature PDF plots along with their fitting are given in Supplementary Fig. 18.

Computational details. We performed first-principles density functional theory (DFT) calculations using the Vienna Ab-initio Simulation Package (VASP)[17,18] with potentials derived using the projector augmented wave (PAW)[19,20] method. We used the following potentials in our

calculations: Cs ($5s^2$ $5p^6$ $6s^1$), Bi ($5d^{10}$ $6s^2$ $6p^3$), Cl ($3s^2$ $3p^5$) and I ($5s^2$ $5p^5$). The exchange-correlation energies of the electron were approximated using the PBEsol[21] functional within the generalized gradient approximation (GGA).[22] The kinetic energy cut-off was set to 520 eV and a k-point mesh of 9 × 9 × 7 was used for the relaxation of the cell parameters. The optimized lattice parameters (a= 8.18 Å, c= 9.93 Å) agree well with the experimentally values (a= 8.24 Å, c= 10.03 Å). The relaxed structure retains the hexagonal space group (P-3m1, No. 164). The Brillouin zone (BZ) for this unit cell is shown in Supplementary Fig. 19a. We calculated the harmonic phonon dispersion of $Cs_3Bi_2I_6Cl_3$ using the finite-displacement method as implemented in the Phonopy[23] code, where the displaced configurations were generated using a 2×2×2 supercell (containing 112 atoms) of the primitive unit cell that has 14 atoms. For phonon calculations, we used a 4×4×4 k-point mesh for the calculation of the forces. The calculated phonon dispersion exhibits quite a few imaginary phonon branches (Supplementary Fig. 7a), which is common in the family of inorganic halide perovskites.[24] The imaginary phonon mode at Γ exhibits a double-well potential energy surface (Supplementary Fig. 7b). To stabilize these imaginary phonons, we nudged the atoms in the primitive unit cell with the eigenvectors of the imaginary phonon mode (-19 cm^{-1}) at the Γ point in the Brillouin zone and relaxed the crystal structure of the unit cell which lowers the symmetry to triclinic (space group: P1, No.1). The relaxed structure was then utilized to calculate the harmonic phonon dispersion (Fig. 3d), phonon density of states (Fig. 3e) and mode Grüneisen parameter (Fig. 3c). The BZ for this triclinic unit cell is shown in Supplementary Fig. 19b. Most of the imaginary phonon modes got stabilized in the phonon dispersion of the relaxed structure (Fig. 3d). We determined the mode Grüneisen parameters of $Cs_3Bi_2I_6Cl_3$ using a finite difference method, where we have calculated the phonon frequencies of the compound at two different volumes ($1.02V_0$ and $0.98V_0$, V_0 being the equilibrium unit cell volume) and utilized the formula $\gamma_{q\nu} = -\frac{dln\omega_{q\nu}}{dlnV}$, where $\gamma_{q\nu}$, V and $\omega_{q\nu}$ denote Grüneisen parameter, unit cell volume, and frequency of a phonon mode at wavevector q for branch ν, respectively. To estimate the elastic properties of $Cs_3Bi_2I_6Cl_3$, we calculated its bulk (B) and shear (G) moduli using Voigt's formula[25] utilizing the elastic tensor obtained from VASP. We have analyzed the chemical bonding of the compound using the crystal orbital Hamilton population (COHP) method the LOBSTER code.[26,27] COHP is a method which partitions electronic energy of bands into pairwise orbital or atomic interactions, identifying their chemical character as bonding, anti-bonding or non-bonding.[26,27] We have

analyzed the symmetry of the Raman modes (Supplementary Fig. 13) using the phonons calculated in Supplementary Fig. 7.

We have also tried to stabilize the unstable phonon modes by applying hydrostatic pressure or isotropic strain. We applied 6% compressive isotropic volume strain on the unit cell of $Cs_3Bi_2I_6Cl_3$ and relaxed its internal coordinates of the compound using DFT calculations. We calculated the harmonic dispersion of this compressed and relaxed unit cell which shows partial stabilization of the unstable phonon modes (Supplementary Fig. 20).

We have also checked the effect of spin-orbit coupling (SOC) on electronic structure and phonon dispersion. The electronic structures of $Cs_3Bi_2I_6Cl_3$ without and with the inclusion of the SOC reveals that SOC reduces the band gap from 1.94 eV (Supplementary Fig. 21a) to 1.31 eV (Supplementary Fig. 21b) and lifts degeneracy of the bands along low symmetry directions. On the other hand, SOC affects only negligibly the phonon dispersion, and it does not help in stabilizing the unstable phonon modes (Supplementary Figs. 21c, d).

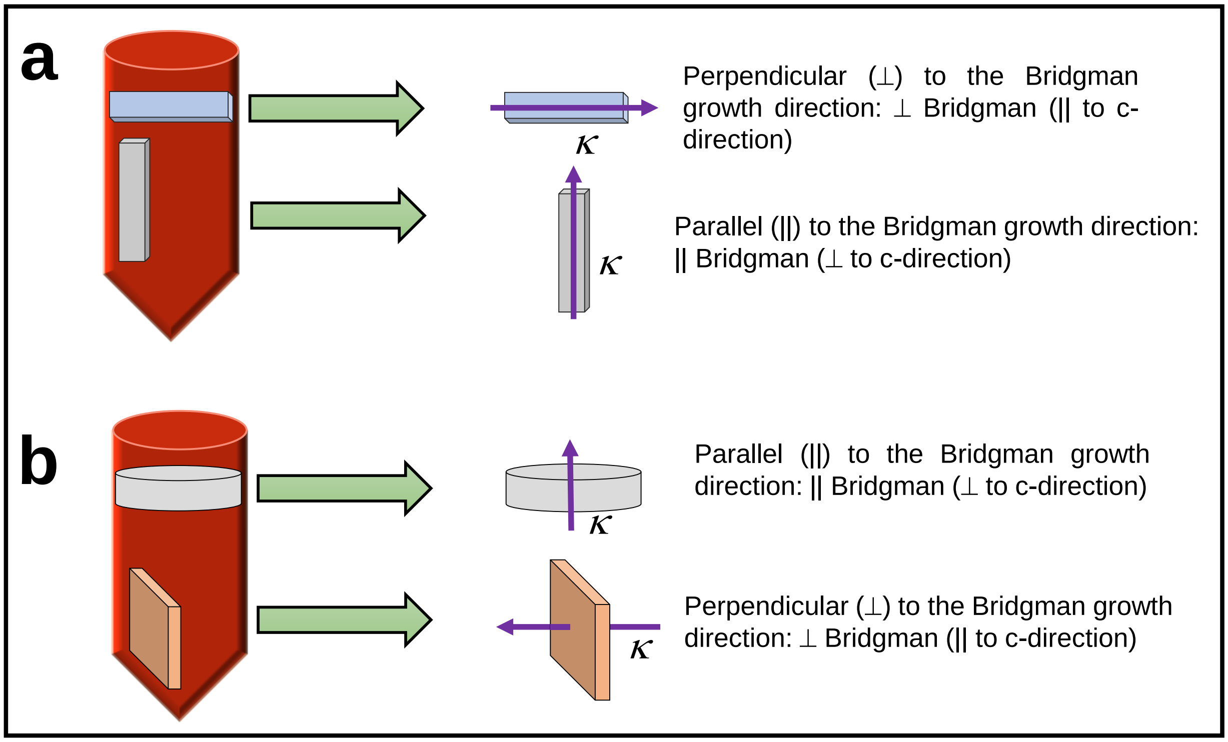

Supplementary Fig. 1. Schematic of $Cs_3Bi_2I_6Cl_3$ single crystal cut. A schematic exhibiting the cut along different directions and thermal conductivity measurement directions for the Bridgman grown $Cs_3Bi_2I_6Cl_3$ single crystal when measured in (**a**) PPMS (2-300 K) and (**b**) LFA-457 (300-400 K).

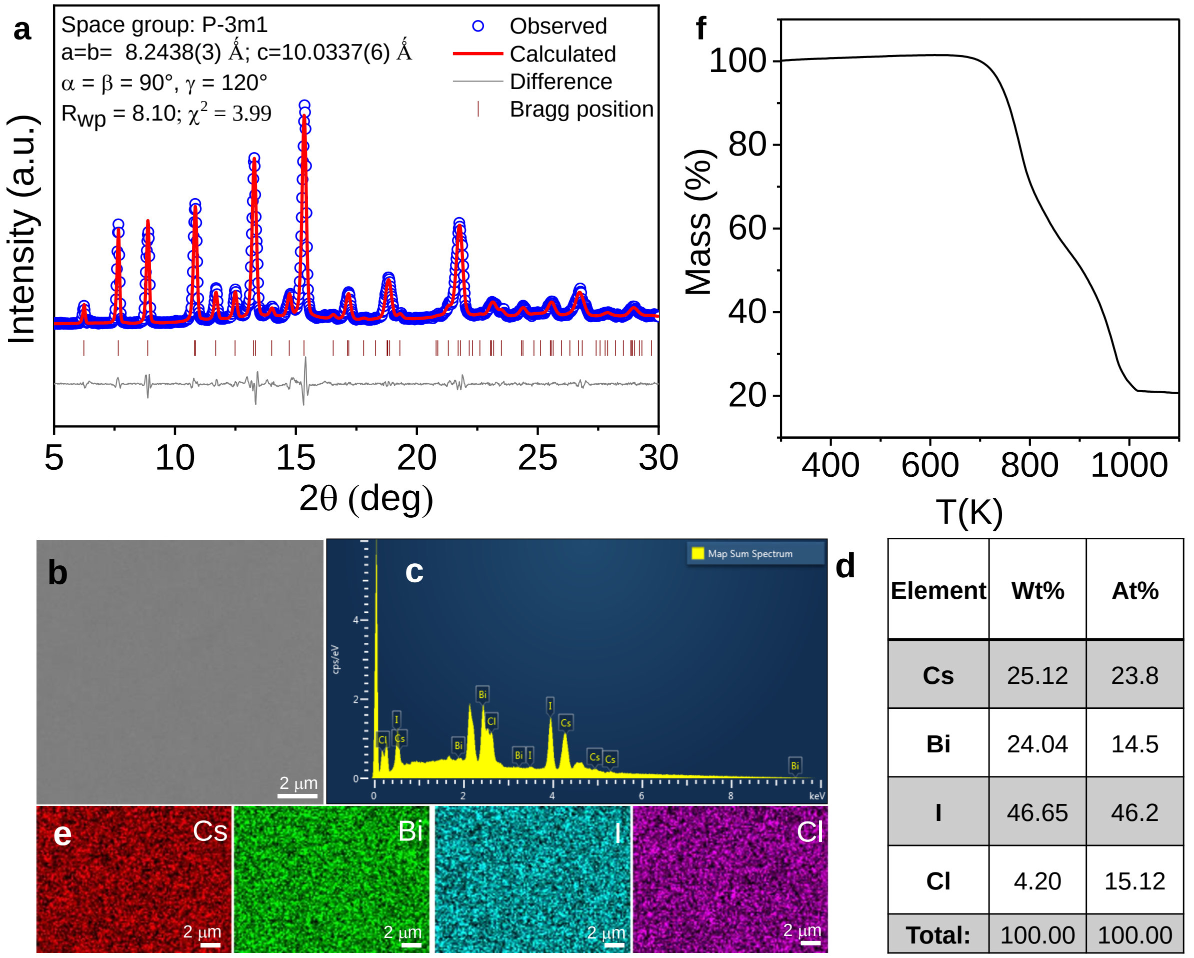

Element	Wt%	At%
Cs	25.12	23.8
Bi	24.04	14.5
I	46.65	46.2
Cl	4.20	15.12
Total:	100.00	100.00

Supplementary Fig. 2. Characterization of $Cs_3Bi_2I_6Cl_3$. (**a**) Refinement of the synchrotron (λ = 0.7762 Å) PXRD data of $Cs_3Bi_2I_6Cl_3$**.** FESEM-BSE image for the Bridgman grown $Cs_3Bi_2I_6Cl_3$ single crystal in (**b**) with corresponding energy-dispersive X-ray (EDX) spectra in (**c**). (**d**) weight and atomic percentage of Cs, Bi, I and Cl atoms. (**e**) EDX elemental color mapping for Cs, Bi, I and Cl for the area in (**b**)**.** (**f**) TGA profile of $Cs_3Bi_2I_6Cl_3$ in N_2 atmosphere.

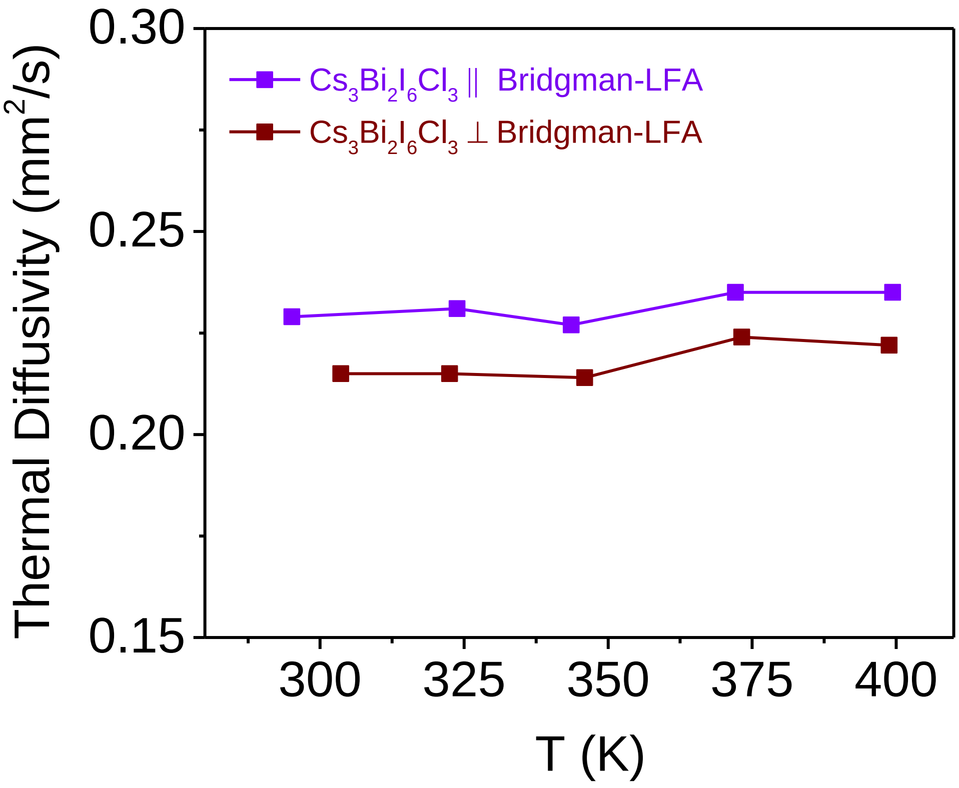

Supplementary Fig. 3. Thermal diffusivity. Temperature-dependent thermal diffusivity (D) of $Cs_3Bi_2I_6Cl_3$.

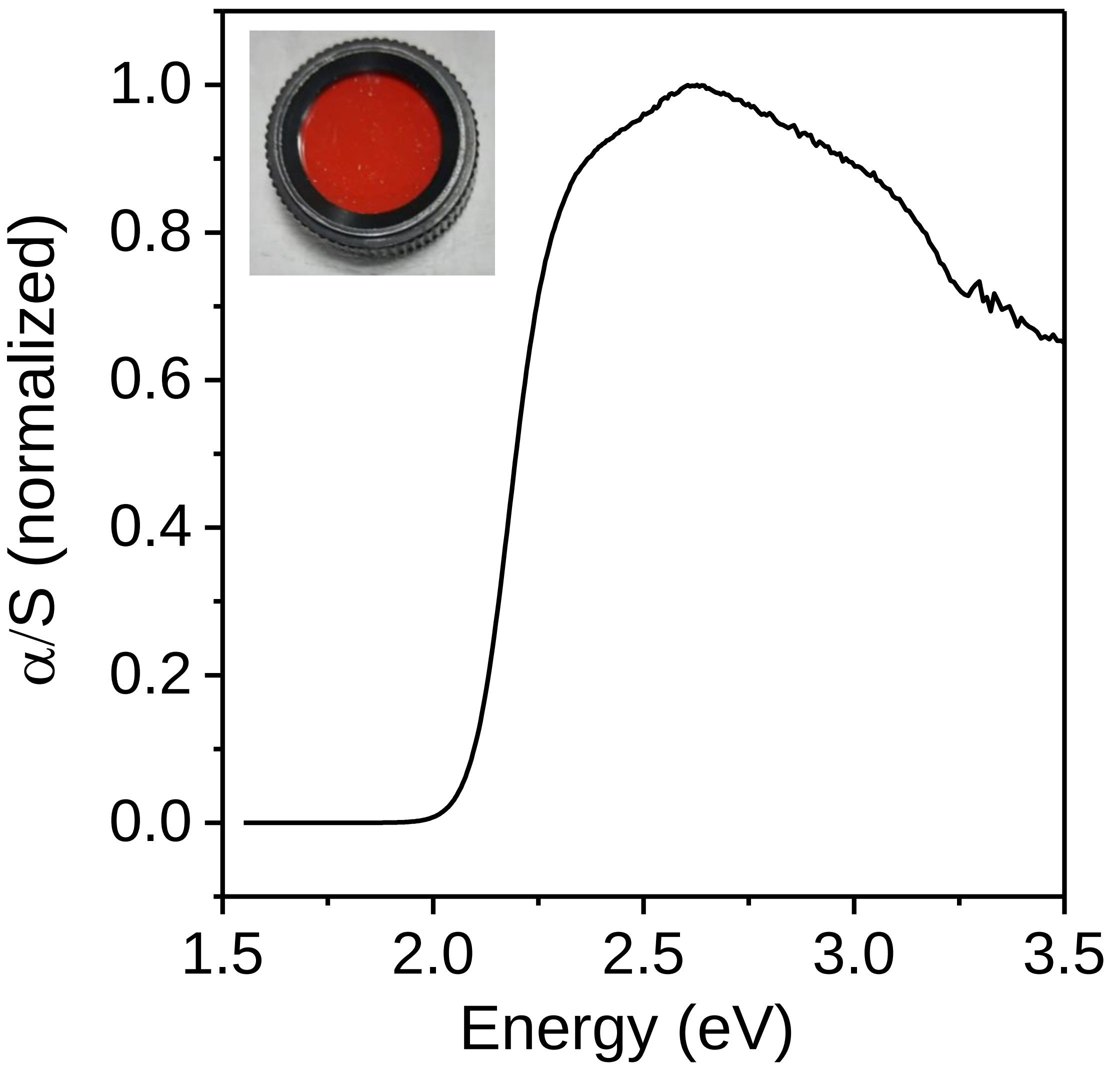

Supplementary Fig. 4. Optical bandgap. Optical absorption spectra of $Cs_3Bi_2I_6Cl_3$.

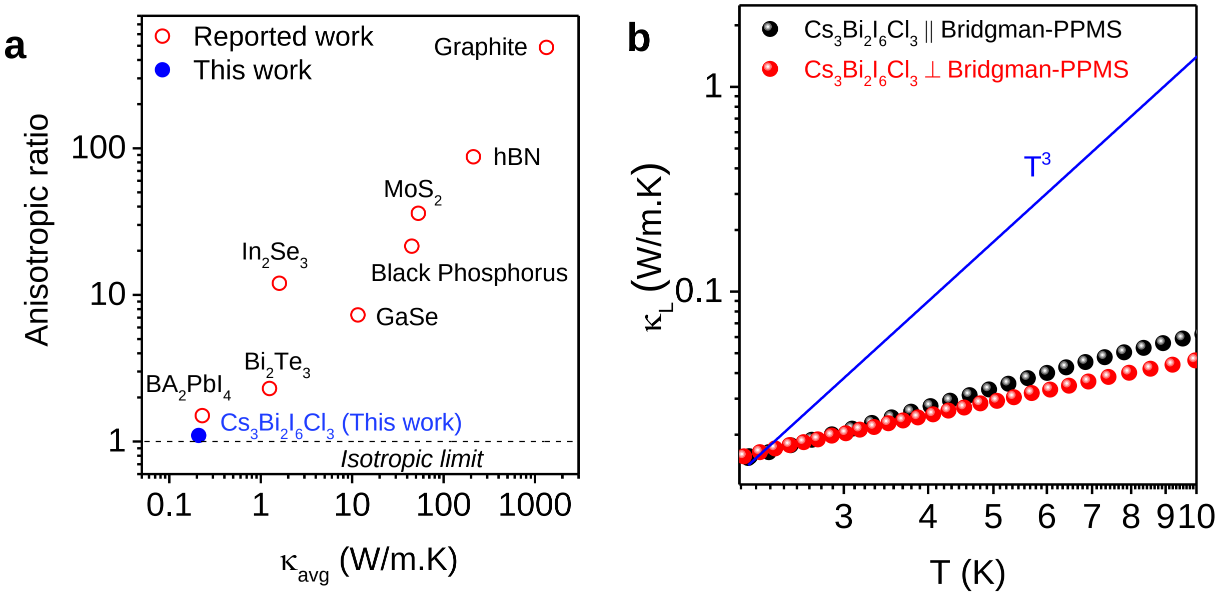

Supplementary Fig. 5. Anisotropic thermal conductivity ratio and low temperature thermal conductivity. (**a**) Comparison plot of anisotropic ratio (defined as $\kappa_L(\perp c)/\ \kappa_L(\|c)$ *vs.* average thermal conductivity ($\kappa_{avg} = [\kappa_L(\perp c)+\kappa_L(\|c)]/2$) of $Cs_3Bi_2I_6Cl_3$ single crystals with other layered single crystals at room temperature. References in the plot are graphite,[28] hexagonal boron nitride (h-BN),[29] MoS_2,[30] black phosphorus,[31] In_2Se_3,[32] GaSe,[28] Bi_2Te_3,[33] and BA_2PbI_4.[34] (**b**) Low temperature thermal conductivity (<10 K) of $Cs_3Bi_2I_6Cl_3$ largely deviates from T^3 dependency.

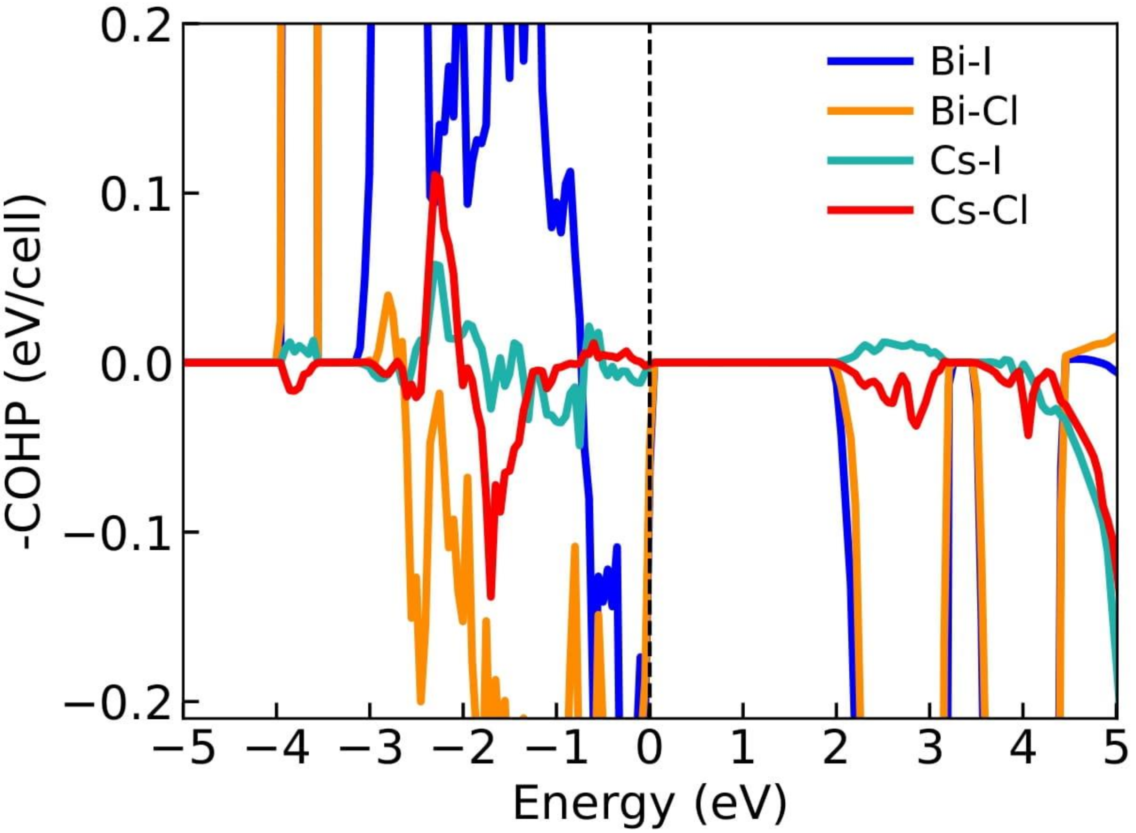

Supplementary Fig. 6. Crystal orbital Hamilton population of $Cs_3Bi_2I_6Cl_3$. Enlarged version (-0.2- 0.2 eV/cell) of crystal orbital Hamilton population (COHP) analysis of $Cs_3Bi_2I_6Cl_3$.

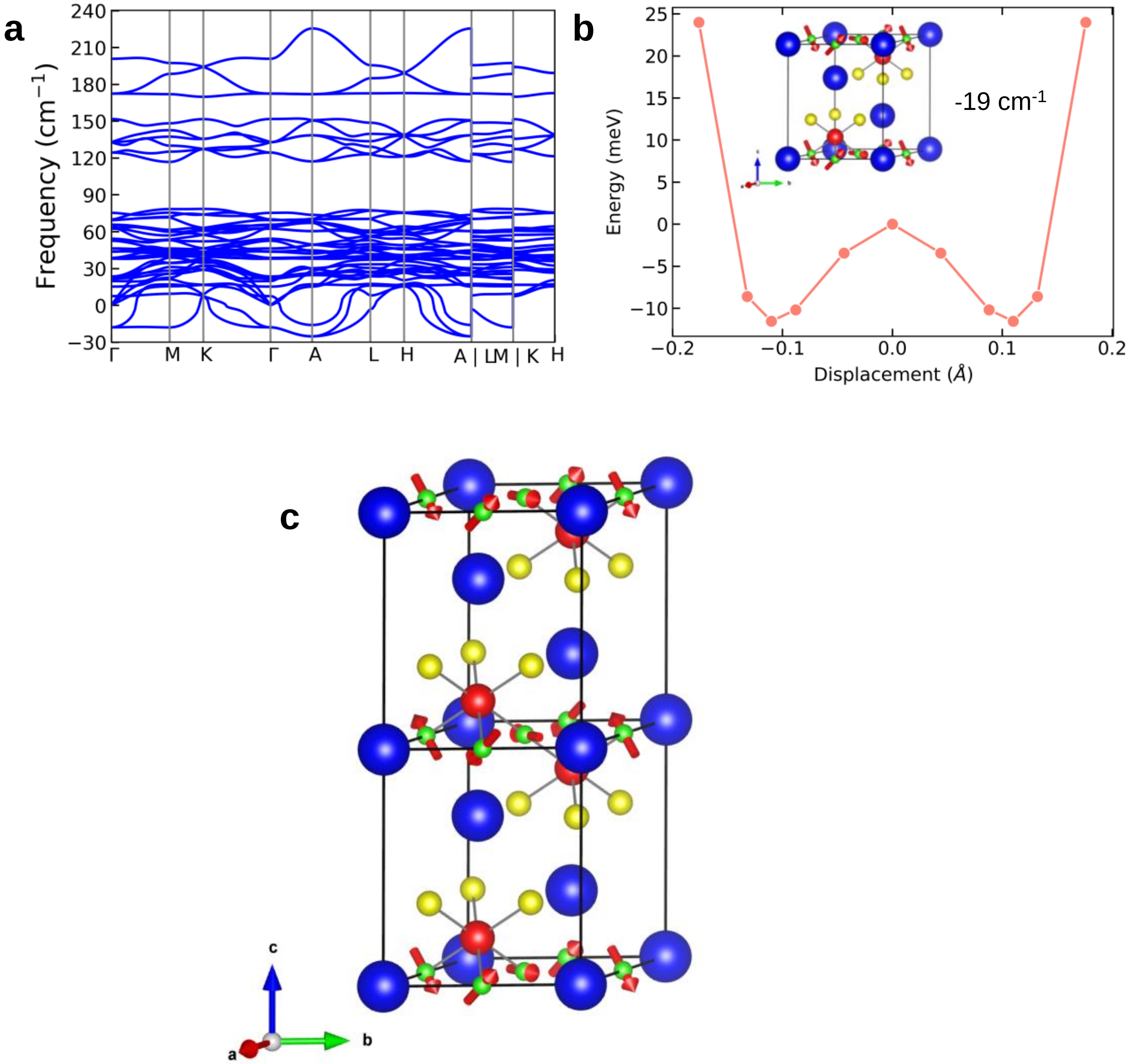

Supplementary Fig. 7. Phonon dispersion of $Cs_3Bi_2I_6Cl_3$. (**a**) Phonon dispersion of the original primitive unit cell of $Cs_3Bi_2I_6Cl_3$ and (**b**) the double-well potential energy of the imaginary phonon mode at Γ point that appear at -19 cm^{-1}. Inset shows the visualization of the eigenvector. (**c**) The visualization of the eigenvector of the lowest frequency (-25 cm^{-1}) phonon mode at A-point in a 1x1x2 supercell. Transverse acoustic (TA) phonons hybridize with the lowest frequency torsional phonon modes (-25 cm^{-1}) at the A-point strongly suppressing TA phonons and reducing the speed of sound. Cs, Bi, I, and Cl atoms are denoted by the blue, red, yellow, and green spheres, respectively.

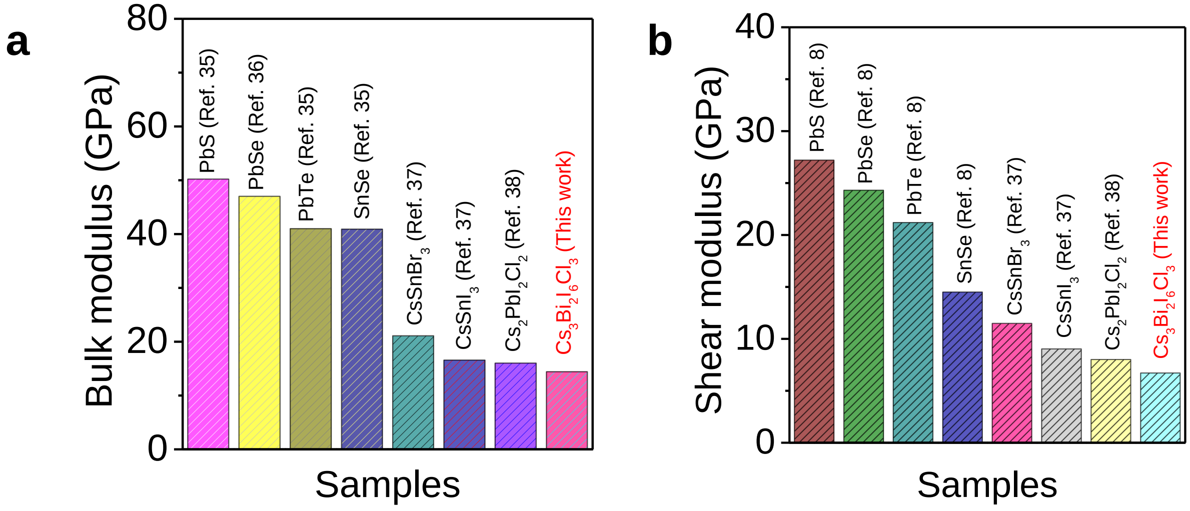

Supplementary Fig. 8. **Elastic modulus.** Comparison of the **(a)** bulk modulus[35-38] and **(b)** shear modulus[8,37,38] of $Cs_3Bi_2I_6Cl_3$ with several well-known low thermal conductive metal chalcogenides and other all-inorganic metal halide perovskites.

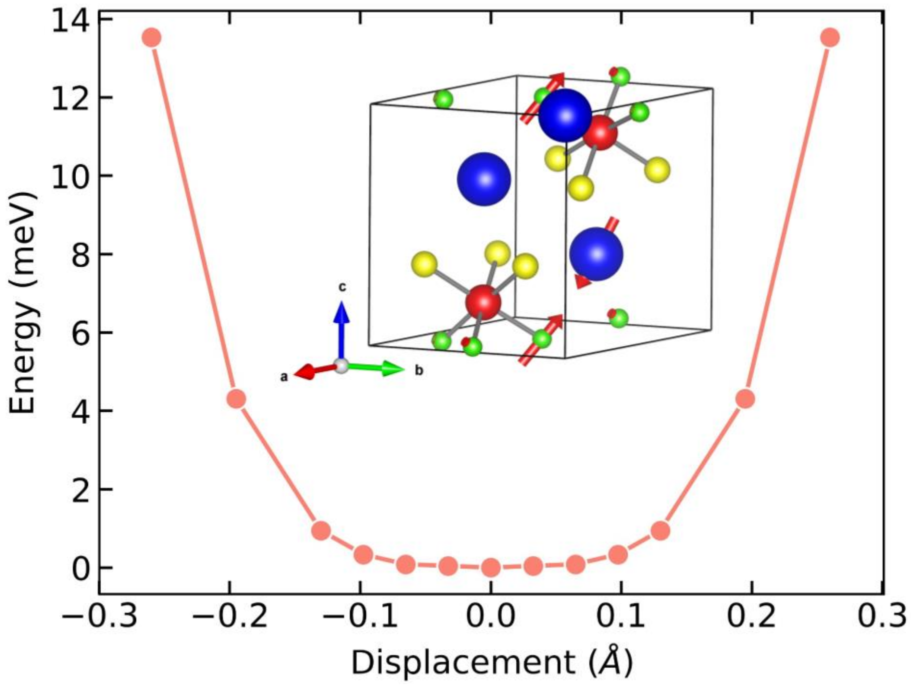

Supplementary Fig. 9. Potential energy diagram. Potential energy of the imaginary phonon mode at Γ point that appears at -12 cm^{-1} in fig. 3d. Inset shows the visualization of the eigenvector. Cs, Bi, I, and Cl atoms are denoted by the blue, red, yellow, and green spheres, respectively.

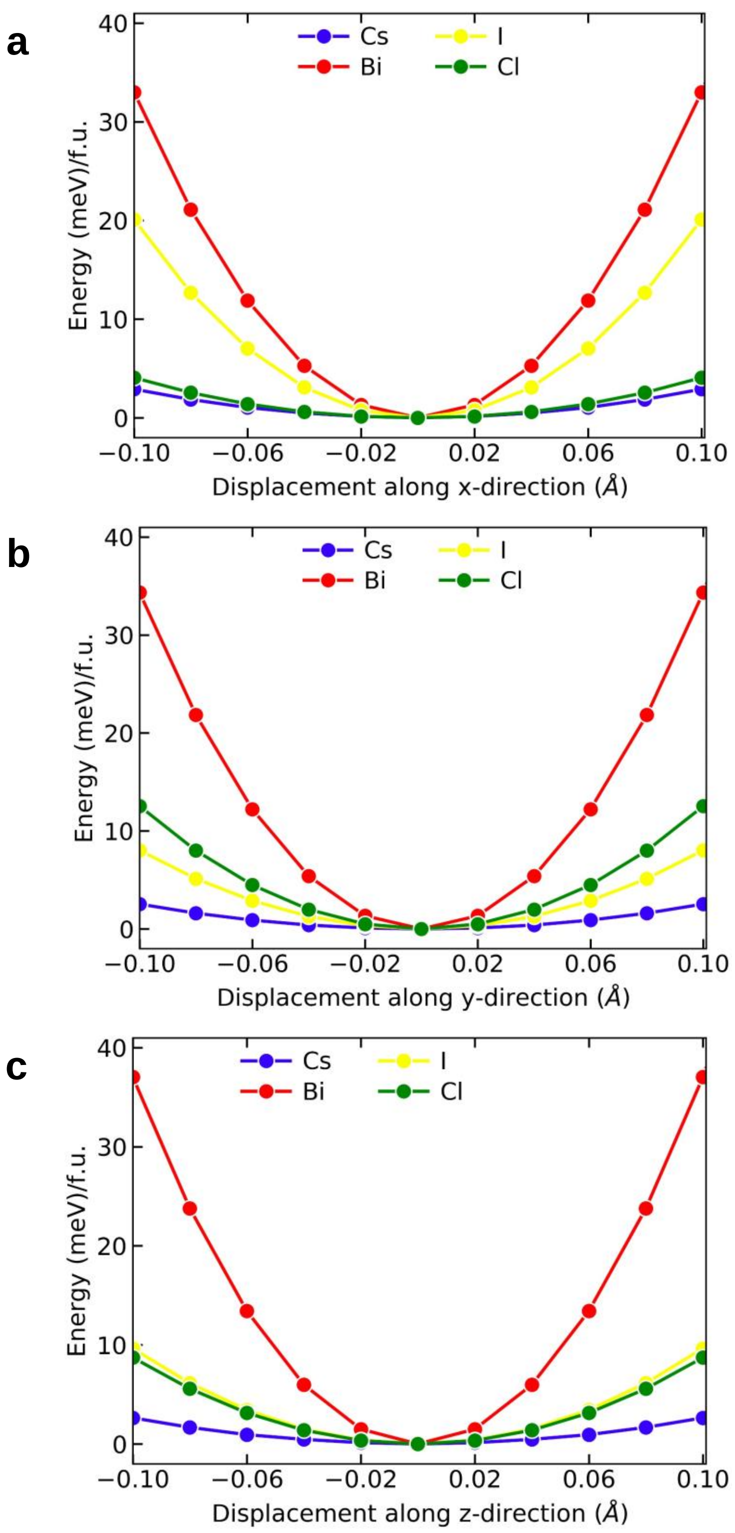

Supplementary Fig. 10. **Potential energy diagram.** Potential energy vs. displacement plot of $Cs_3Bi_2I_6Cl_3$ along **(a)** x-direction, **(b)** y-direction and **(c)** z-direction.

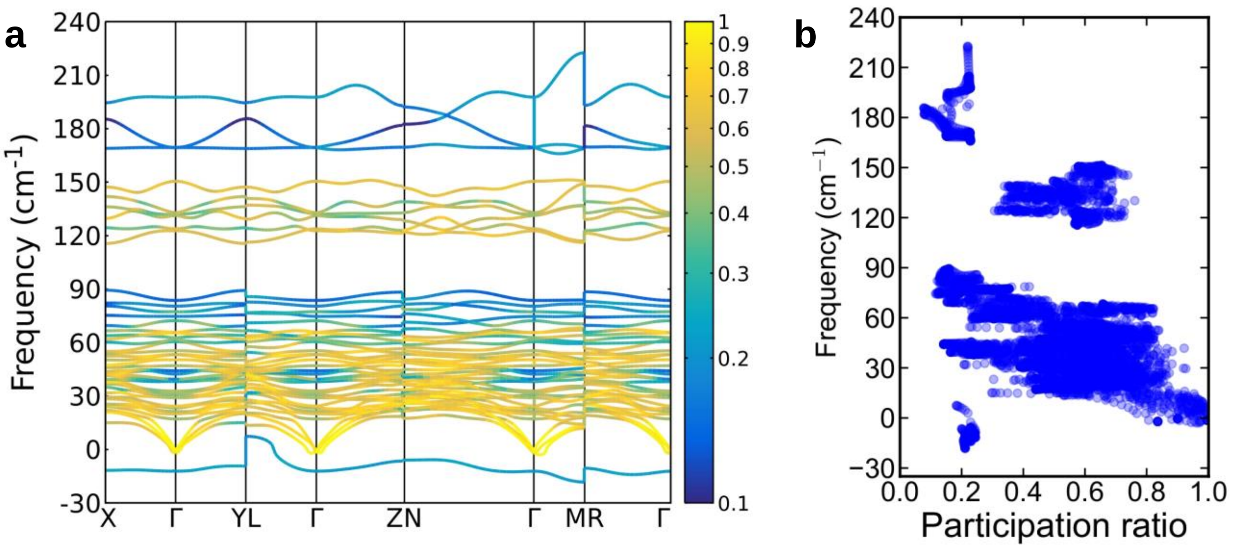

Supplementary Fig. 11. Participation ratio. (a) Calculated participation ratio (PR)[39] for the phonon modes of $Cs_3Bi_2I_6Cl_3$. Lower value (~0-0.3) of PR indicates more localized phonon modes. **(b)** PR vs. frequency shows the presence of diffuson modes (0.1 <PR <0.6) in $Cs_3Bi_2I_6Cl_3$.[40-42]

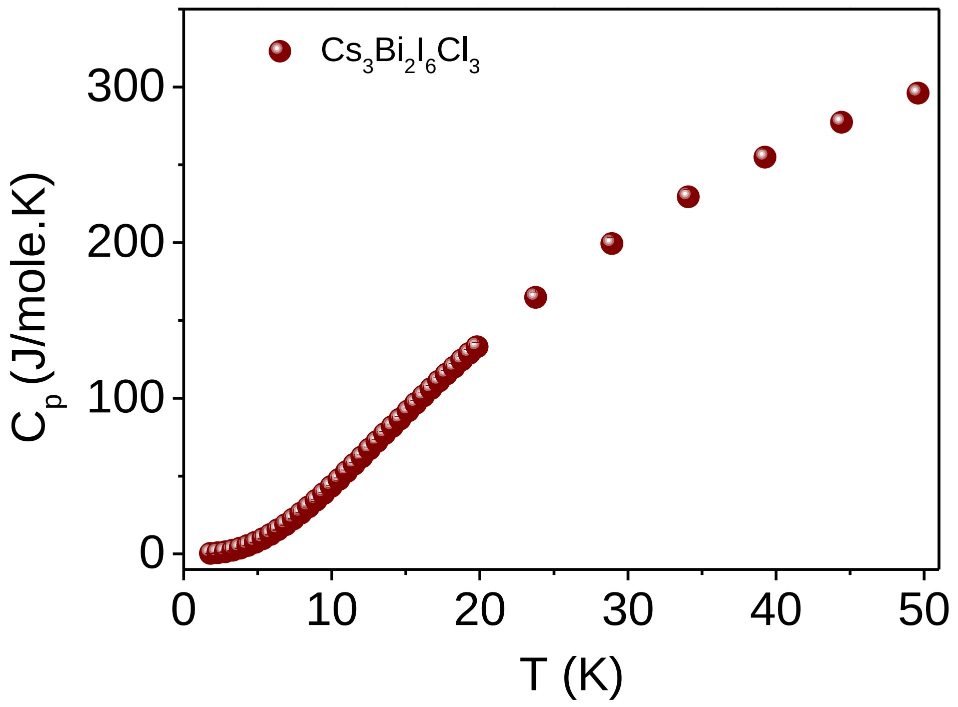

Supplementary Fig. 12. Heat capacity. Low temperature heat capacity (C_p) of $Cs_3Bi_2I_6Cl_3$.

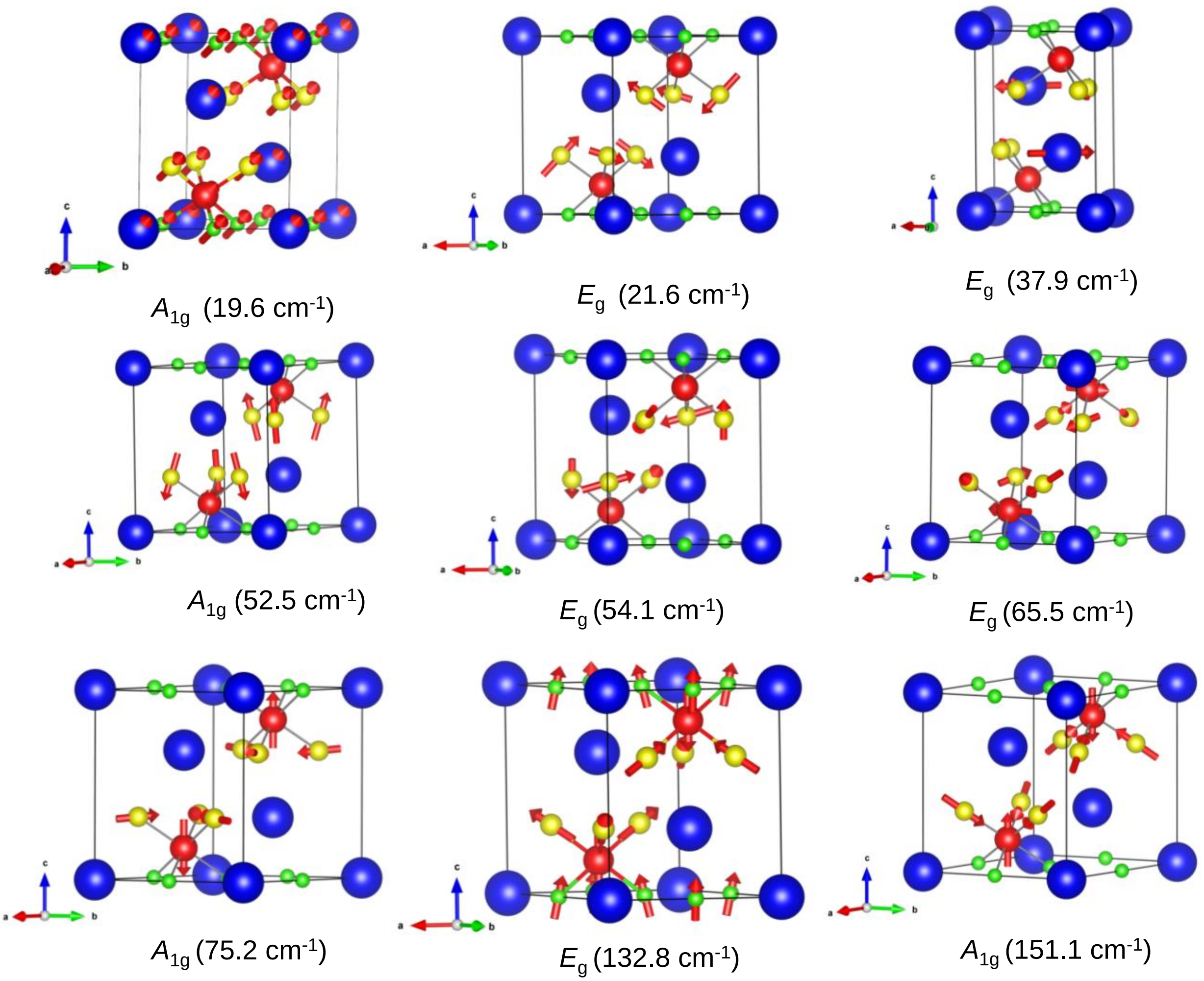

Supplementary Fig. 13. **Eigenvector visualizations.** Eigenvector visualizations of Raman active modes at Γ-point of $Cs_3Bi_2I_6Cl_3$. Cs, Bi, I, and Cl atoms are denoted by the blue, red, yellow, and green spheres, respectively.

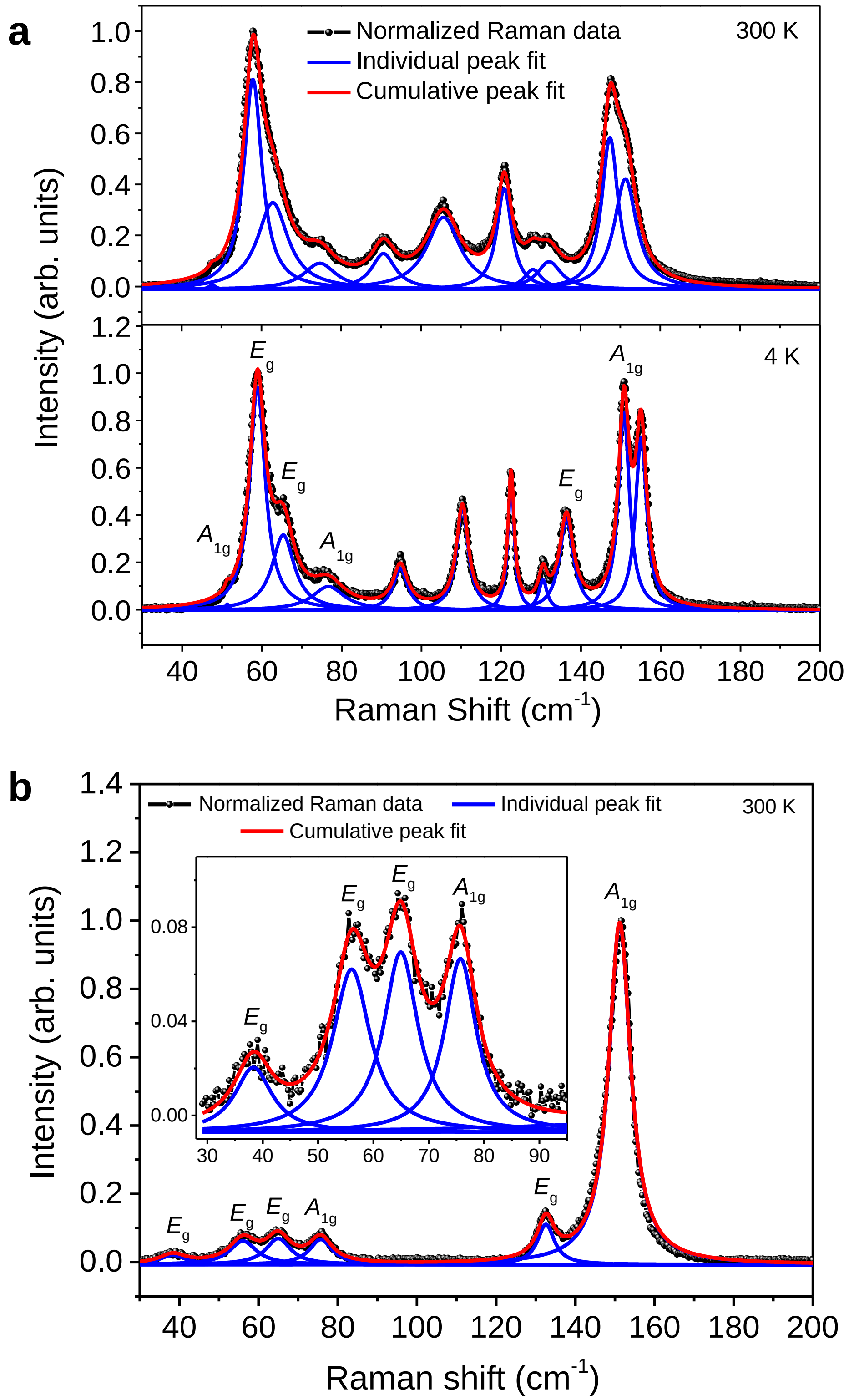

Supplementary Fig. 14. Raman spectra. (a) Raman spectra of $Cs_3Bi_2I_6Cl_3$ at 4 K and 300 K collected using a 785 nm laser. **(b)** Room temperature Raman spectra of $Cs_3Bi_2I_6Cl_3$ measured using a 633 nm laser.

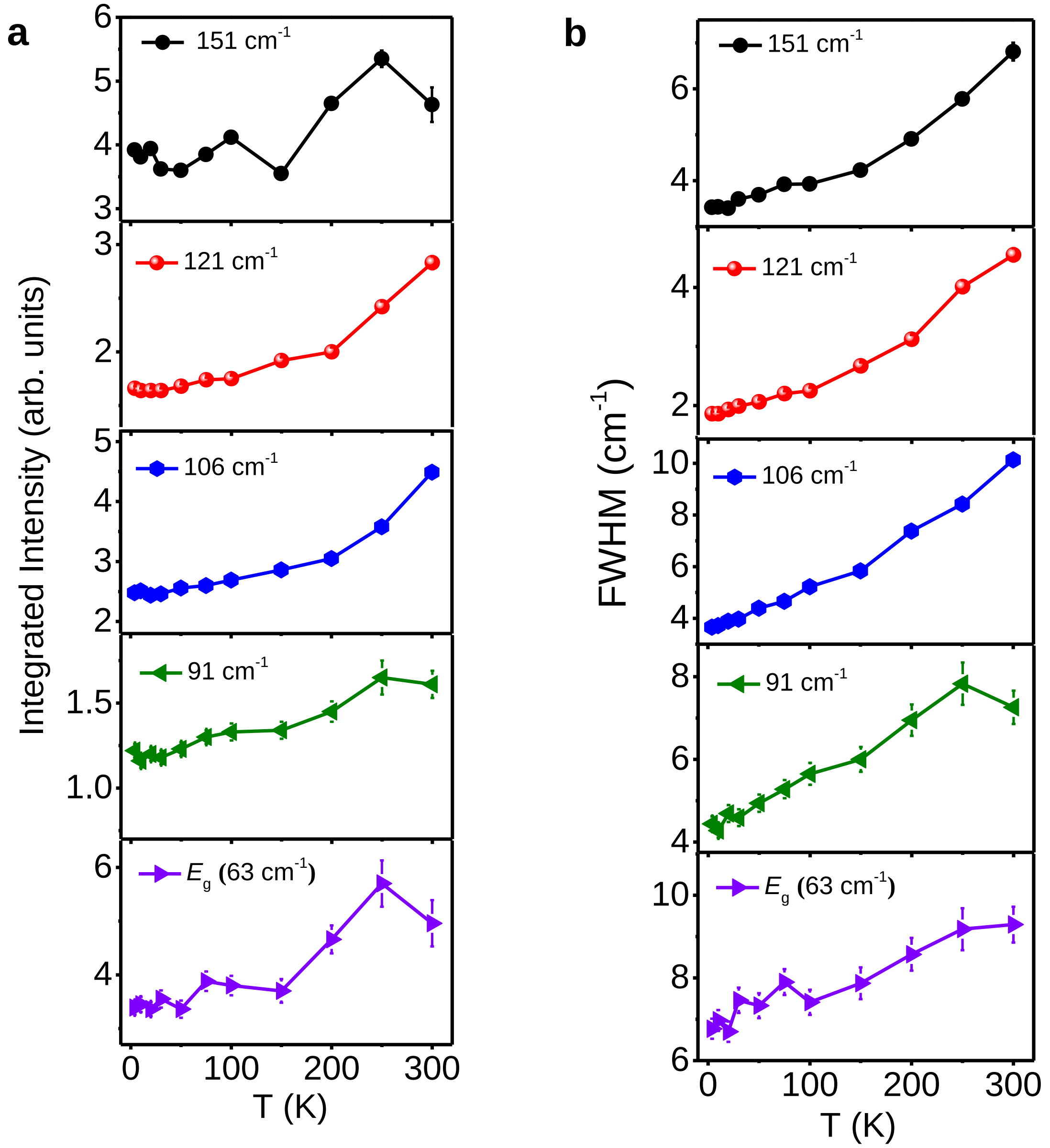

Supplementary Fig. 15. Temperature-dependent integrated intensity and FWHM. Temperature-dependent (**a**) integrated intensity and (**b**) FWHM of different Raman peaks of $Cs_3Bi_2I_6Cl_3$.

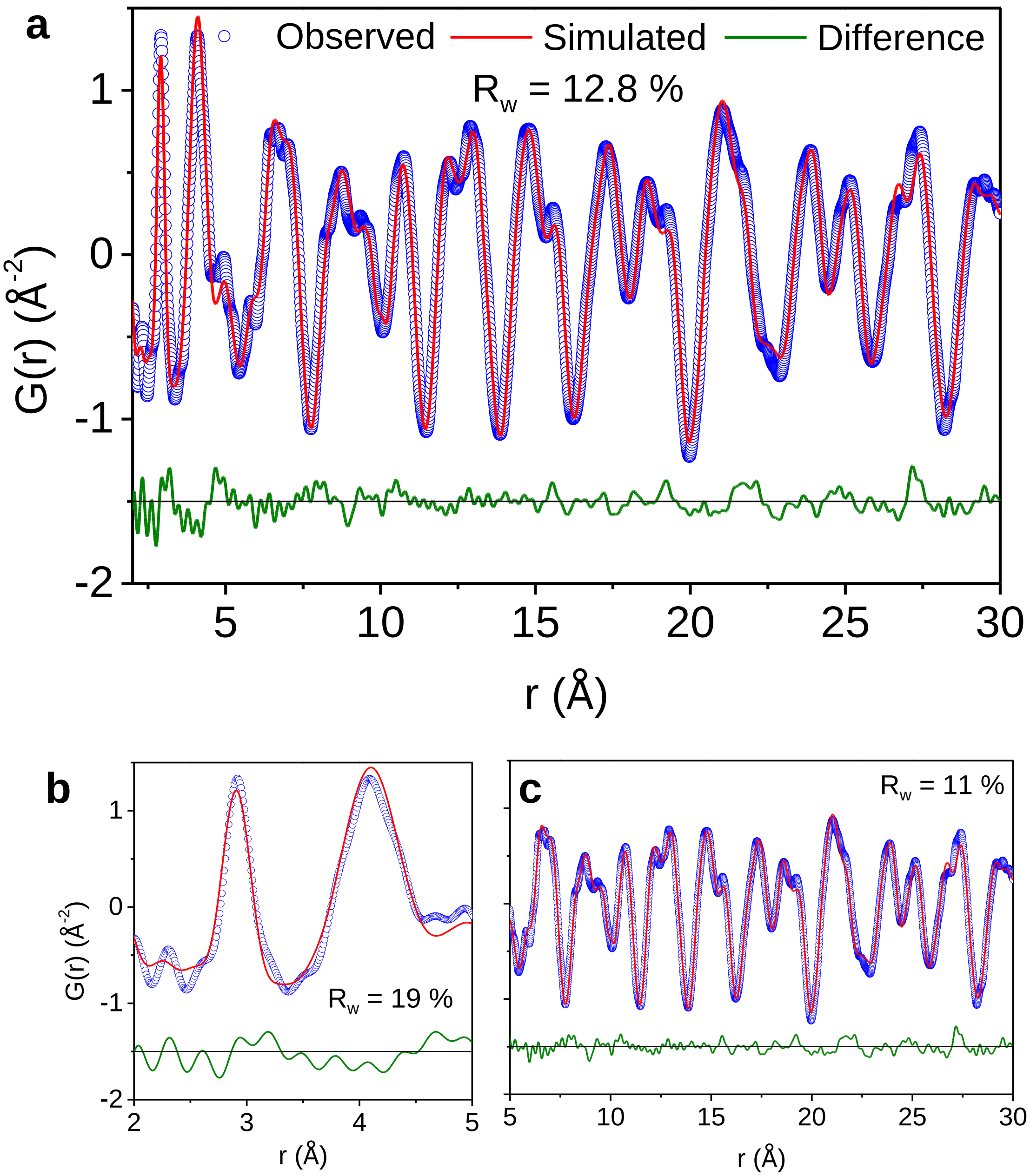

Supplementary Fig. 16. Synchrotron X-ray PDF. (a) Synchrotron X-ray PDF data and fitting only with thermal parameter refinement of $Cs_3Bi_2I_6Cl_3$ at 300 K. **(b)** Fitting of local structure (2-5 Å) and **(c)** average structure (5-30 Å) of $Cs_3Bi_2I_6Cl_3$ using only thermal parameters refinement.

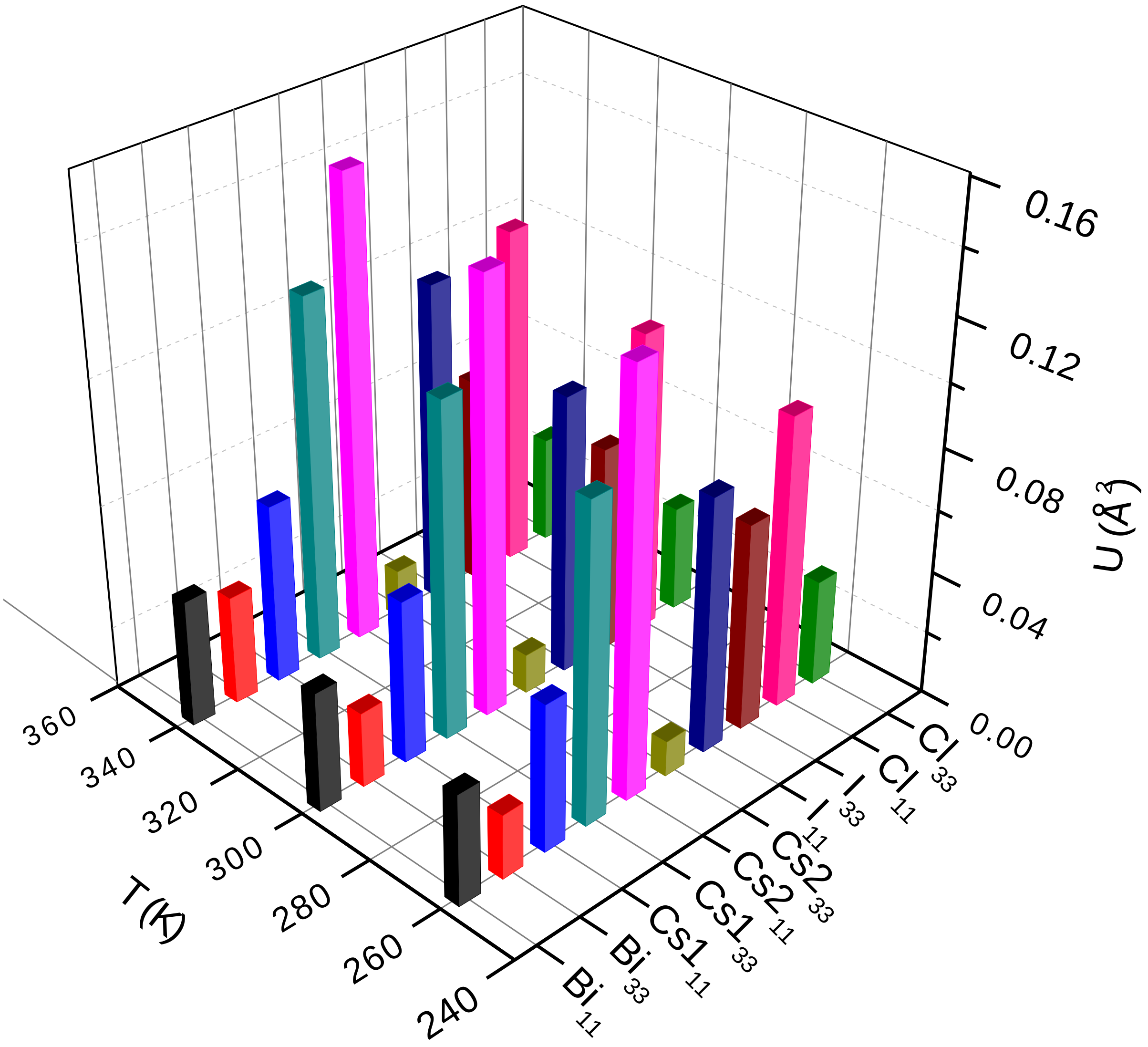

Supplementary Fig. 17. Atomic displacement parameter. Temperature dependent atomic displacement parameter (ADP) values of different atoms of $Cs_3Bi_2I_6Cl_3$ along crystallographic a and b-axis (given by A_{11}) and c-axis (A_{33}). A = Bi, Cs1, Cs2, I, Cl.

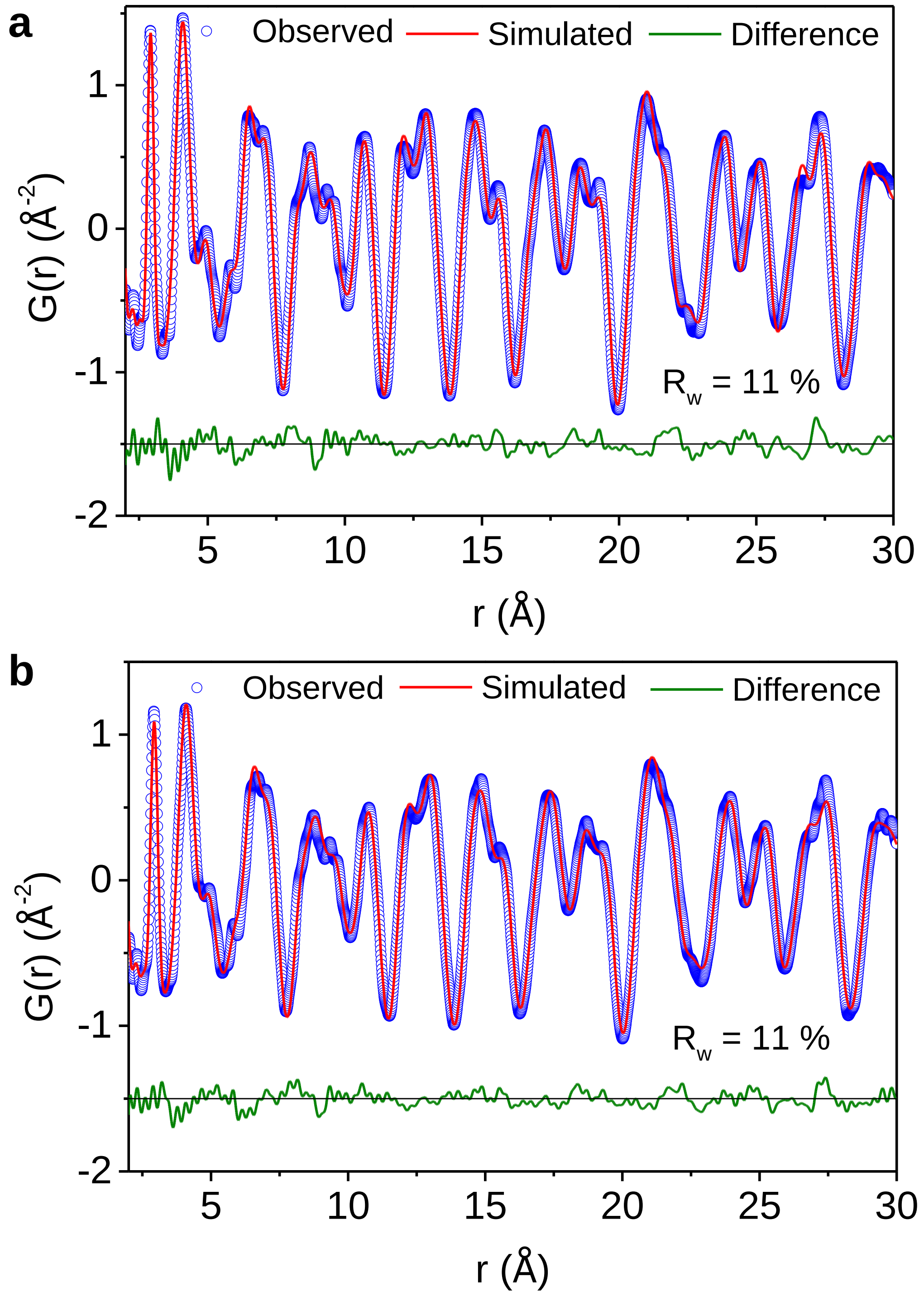

Supplementary Fig. 18. Synchrotron X-ray PDF. Synchrotron X-ray PDF data of $Cs_3Bi_2I_6Cl_3$ fitted using *P*-3*m*1 space group (ambient crystal structure) with all atomic positions and thermal parameters refined at **(a)** 260 K and **(b)** 340 K.

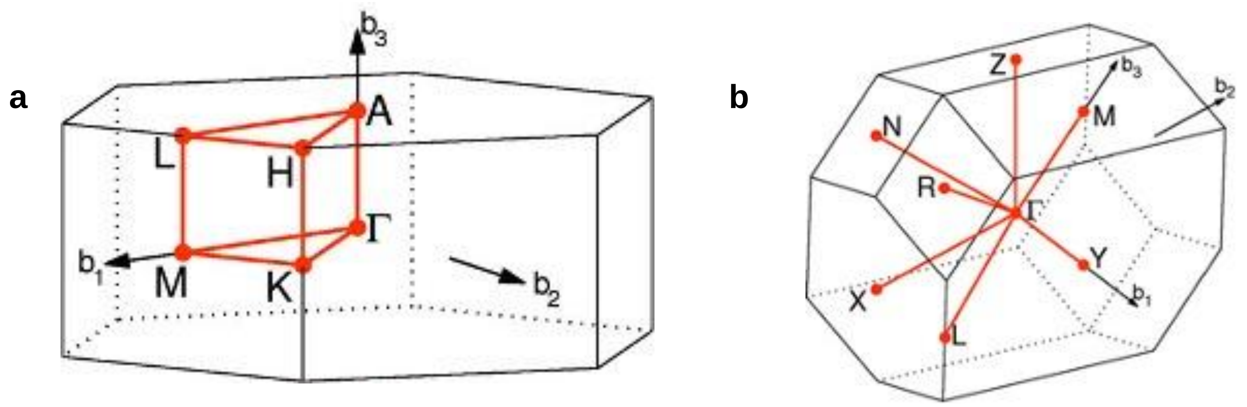

Supplementary Fig. 19. Brillouin zones. Brillouin zones of the (**a**) hexagonal and (**b**) triclinic unit cells of $Cs_3Bi_2I_6Cl_3$, with high-symmetry points. Figures are taken from reference.[43]

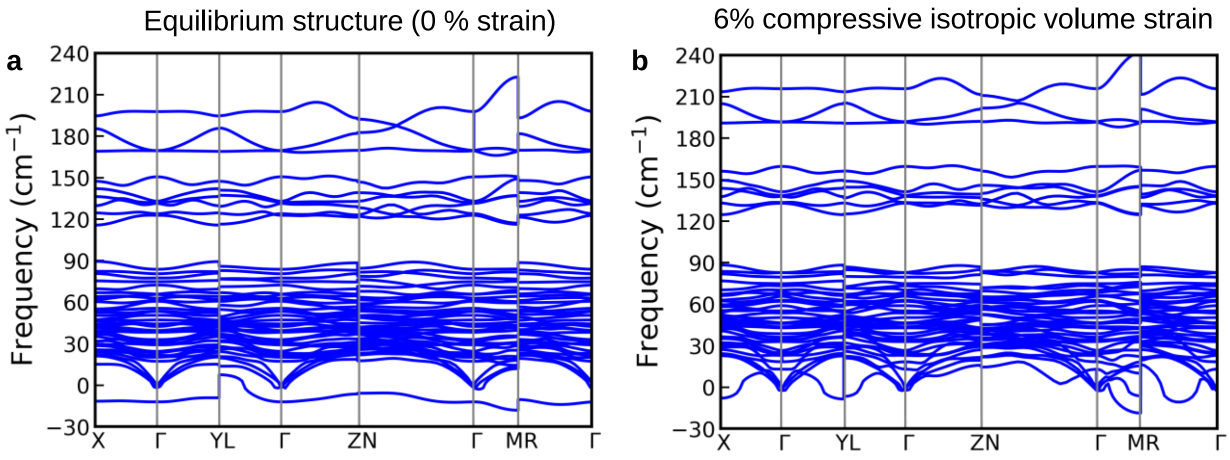

Supplementary Fig. 20. Phonon dispersions. Phonon dispersion of $Cs_3Bi_2I_6Cl_3$ for the **(a)** equilibrium crystal structure (i.e., 0 % strain) and **(b)** for the strained crystal structure, where volume is compressed isotropically by 6%.

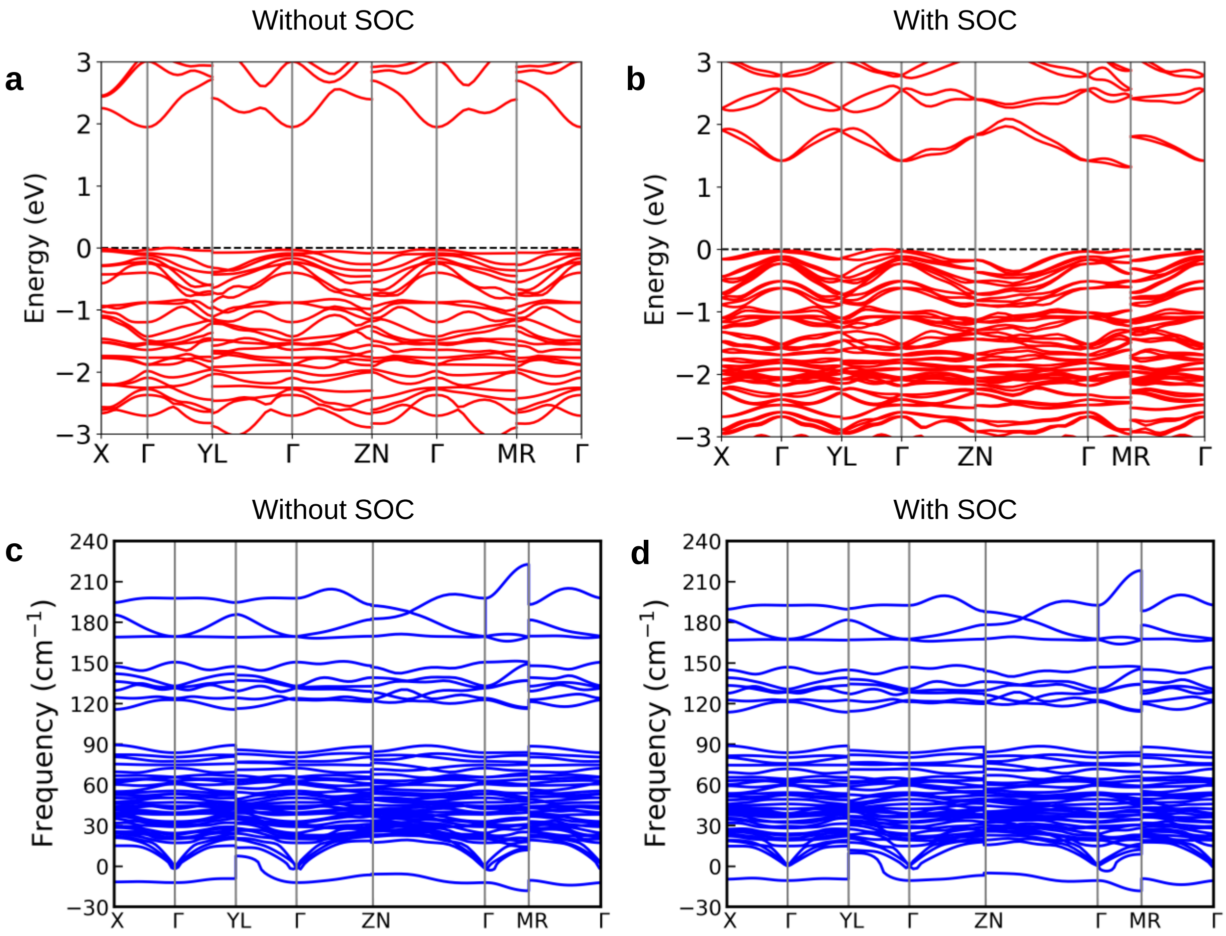

Supplementary Fig. 21. Electronic structures and phonon dispersions. Electronic structures **(a, b)** and phonon dispersions **(c, d)** of $Cs_3Bi_2I_6Cl_3$ calculated without and with the inclusion of spin-orbit coupling (SOC).

Supplementary Table 1. Single crystal data and structure refinement for $Cs_3Bi_2I_6Cl_3$ at 295(2) K.

Empirical formula	$Cs_3Bi_2I_6Cl_3$
Formula weight	1684.44 g/mol
Temperature	295 (2) K
Wavelength	0.71073 Å
Crystal System	Trigonal
Space group	*P*-3*m*1
Unit cell dimensions	a = 8.2985(10) Å; α = 90°
	b = 8.2985(10) Å; β = 90°
	c = 10.1591(18) Å; γ = 120°
Volume	605.88(18) $Å^3$
Z	1
Calculated density	4.617 g/cm^3
F(000)	700
Crystal size	0.19×0.18×0.12 mm^3
$\theta_{min,max}$	2.005, 25.448
$h_{min,max}$<br>$k_{min,max}$<br>$l_{min,max}$	-10, 10<br>-9, 10<br>-12, 11
Absorption coefficient	26.896 mm^{-1}
Reflections collected	3914
Unique reflections/ No. parameters	458/19
Goodness-of-fit	1.087
Final R indices (I>2σ (I))	R_{obs} = 0.0728, wR_{obs} = 0.1732
R indices (all data)	R_{all} = 0.1006, wR_{all} = 0.1885
CCDC Number	2150864

Supplementary Table 2. Site occupancy and anisotropic displacement parameters for $Cs_3Bi_2I_6Cl_3$ single crystal at 295 (2) K.

**Atom**	**Occupancy**	$\mathbf{U_{11}}$	$\mathbf{U_{22}}$	$\mathbf{U_{33}}$	$^{*}\mathbf{U_{equiv}}$
**Cs1**	1.030(9)	0.125(3)	0.125(3)	0.084(4)	0.0988(13)
**Cs2**	1.010(8)	0.0840(16)	0.0840(16)	0.128(3)	0.111(2)
**Bi**	1.030(9)	0.0576(8)	0.0576(8)	0.0807(14)	0.0653(8)
**I**	0.990(9)	0.0944(15)	0.0709(14)	0.117(2)	0.0967(10)
**Cl**	1.020(8)	0.101(6)	0.101(6)	0.056(5)	0.074(3)

$^{*}U_{equiv}$ is defined as one-third of the trace of the orthogonalized U_{ij} tensor.

Supplementary Table 3. Experimental sound velocities, minimum thermal conductivity (κ_{min}), diffuson thermal conductivity (κ_{diff}), Poisson ratio (ν_p) and Grüneisen parameters (γ) of $Cs_3Bi_2I_6Cl_3$.

Sample	Longitudinal sound velocity (m/s)	Transverse sound velocity (m/s)	Average sound velocity (m/s)	κ_{min} (W/m·K)	κ_{diff} (W/m·K)	ν_p	γ
$Cs_3Bi_2I_6Cl_3$ (⊥ Bridgman)	2116	954	1076	0.18	0.11	0.37	2.3
$Cs_3Bi_2I_6Cl_3$ (‖ Bridgman)	2136	1028	1156	0.19	0.12	0.35	2.1

Supplementary Table 4. Magnitude of elastic moduli (bulk and shear modulus) of $Cs_3Bi_2I_6Cl_3$ estimated from theory and experimental sound velocity.

**Elastic moduli**	**Theoretical calculation (GPa)**	**Experimental estimation (GPa)**	
		$Cs_3Bi_2I_6Cl_3$ (⊥ Bridgman)	$Cs_3Bi_2I_6Cl_3$ (‖ Bridgman)
Bulk modulus	14.4	15.2	14.7
Shear modulus	6.7	4.2	4.9

Supplementary Table 5. Parameters obtained from the fitting of low-temperature C_p/T vs. T^2 data of $Cs_3Bi_2I_6Cl_3$ using a Debye-Einstein model with three Einstein oscillators.

Parameter	Value
γ (J/mole.K^2)	0.15926
θ_D (K)	82
θ_{E1} (K)	19.2
θ_{E2} (K)	40.9
θ_{E3} (K)	76.9

Supplementary Table 6. Refinement of site occupancy of $Cs_3Bi_2I_6Cl_3$ using synchrotron X-ray PDF data at 300 K.

Atom	Occupancy
**Cs1**	1.000
**Cs2**	1.003
**Bi**	1.001
**I**	0.992
**Cl**	0.957

Supplementary References

1 Sheldrick, G. A short history of SHELX. *Acta. Cryst.* **64**, 112-122 (2008).

2 Sheldrick, G. Crystal structure refinement with SHELXL. *Acta. Cryst.* **71**, 3-8 (2015).

3 Farrugia, L. WinGX suite for small-molecule single-crystal crystallography. *J. Appl. Cryst.* **32**, 837-838 (1999).

4 Rodríguez-Carvajal, J. Recent advances in magnetic structure determination by neutron powder diffraction. *Physica B Condens. Matter* **192**, 55-69 (1993).

5 Cahill, D. G., Watson, S. K. & Pohl, R. O. Lower limit to the thermal conductivity of disordered crystals. *Phys. Rev. B* **46**, 6131-6140 (1992).

6 Agne, M. T., Hanus, R. & Snyder, G. J. Minimum thermal conductivity in the context of diffuson-mediated thermal transport. *Energy Environ. Sci.* **11**, 609-616 (2018).

7 Zhao, L.-D., He, J., Berardan, D., Lin, Y., Li, J.-F., Nan, C.-W. & Dragoe, N. BiCuSeO oxyselenides: new promising thermoelectric materials. *Energy Environ. Sci.* **7**, 2900-2924 (2014).

8 Xiao, Y., Chang, C., Pei, Y., Wu, D., Peng, K., Zhou, X., Gong, S., He, J., Zhang, Y., Zeng, Z. & Zhao, L.-D. Origin of low thermal conductivity in SnSe. *Phys. Rev. B* **94**, 125203 (2016).

9 Pal, K., Park, C. W., Xia, Y., Shen, J. & Wolverton, C. Scale-invariant machine-learning model accelerates the discovery of quaternary chalcogenides with ultralow lattice thermal conductivity. *NPJ Comput. Mater.* **8**, 48 (2022).

10 Jana, M. K., Pal, K., Warankar, A., Mandal, P., Waghmare, U. V. & Biswas, K. Intrinsic Rattler-Induced Low Thermal Conductivity in Zintl Type $TlInTe_2$. *J. Am. Chem. Soc.* **139**, 4350-4353 (2017).

11 Samanta, M., Pal, K., Pal, P., Waghmare, U. V. & Biswas, K. Localized Vibrations of Bi Bilayer Leading to Ultralow Lattice Thermal Conductivity and High Thermoelectric

Performance in Weak Topological Insulator n-Type BiSe. *J. Am. Chem. Soc.* **140**, 5866-5872 (2018).

12 Dippel, A.-C., Liermann, H.-P., Delitz, J. T., Walter, P., Schulte-Schrepping, H., Seeck, O. H. & Franz, H. Beamline P02.1 at PETRA III for high-resolution and high-energy powder diffraction. *J. Synchrotron Rad.* **22**, 675-687 (2015).

13 Proffen, T., Billinge, S. J. L., Egami, T. & Louca, D. Structural analysis of complex materials using the atomic pair distribution function — a practical guide. *Z. Kristallogr. Cryst. Mater.* **218**, 132-143 (2003).

14 Basham, M., Filik, J., Wharmby, M. T., Chang, P. C. Y., El Kassaby, B., Gerring, M., Aishima, J., Levik, K., Pulford, B. C. A., Sikharulidze, I., Sneddon, D., Webber, M., Dhesi, S. S., Maccherozzi, F., Svensson, O., Brockhauser, S., Naray, G. & Ashton, A. W. Data Analysis WorkbeNch (DAWN). *J. Synchrotron Rad.* **22**, 853-858 (2015).

15 Juhas, P., Davis, T., Farrow, C. L. & Billinge, S. J. L. PDFgetX3: a rapid and highly automatable program for processing powder diffraction data into total scattering pair distribution functions. *J. Appl. Crystallogr.* **46**, 560-566 (2013).

16 Farrow, C. L., Juhas, P., Liu, J. W., Bryndin, D., Božin, E. S., Bloch, J., Proffen, T. & Billinge, S. J. L. PDFfit2 and PDFgui: computer programs for studying nanostructure in crystals. *J. Phys. Condens. Matter* **19**, 335219 (2007).

17 Kresse, G. & Furthmüller, J. Efficiency of ab-initio total energy calculations for metals and semiconductors using a plane-wave basis set. *Comput. Mater. Sci.* **6**, 15-50 (1996).

18 Kresse, G. & Furthmüller, J. Efficient iterative schemes for ab initio total-energy calculations using a plane-wave basis set. *Phys. Rev. B* **54**, 11169-11186 (1996).

19 Blöchl, P. E. Projector augmented-wave method. *Phys. Rev. B* **50**, 17953-17979 (1994).

20 Kresse, G. & Joubert, D. From ultrasoft pseudopotentials to the projector augmented-wave method. *Phys. Rev. B* **59**, 1758-1775 (1999).

21 Perdew, J. P., Ruzsinszky, A., Csonka, G. I., Vydrov, O. A., Scuseria, G. E., Constantin, L. A., Zhou, X. & Burke, K. Restoring the Density-Gradient Expansion for Exchange in Solids and Surfaces. *Phys. Rev. Lett.* **100**, 136406 (2008).

22 Perdew, J. P., Burke, K. & Ernzerhof, M. Generalized Gradient Approximation Made Simple. *Phys. Rev. Lett.* **77**, 3865-3868 (1996).

23 Togo, A. & Tanaka, I. First principles phonon calculations in materials science. *Scr. Mater.* **108**, 1-5 (2015).

24 Yang, R. X., Skelton, J. M., da Silva, E. L., Frost, J. M. & Walsh, A. Assessment of dynamic structural instabilities across 24 cubic inorganic halide perovskites. *J. Chem. Phys.* **152**, 024703 (2020).

25 Nye, J. F. *Physical properties of crystals: Their representation by tensors and matrices*. (Oxford University Press, 1957).

26 Deringer, V. L., Tchougréeff, A. L. & Dronskowski, R. Crystal Orbital Hamilton Population (COHP) Analysis As Projected from Plane-Wave Basis Sets. *J. Phys. Chem. A* **115**, 5461-5466 (2011).

27 Dronskowski, R. & Bloechl, P. E. Crystal orbital Hamilton populations (COHP): energy-resolved visualization of chemical bonding in solids based on density-functional calculations. *J. Phys. Chem.* **97**, 8617-8624 (1993).

28 McKinney, R., Gorai, P., Toberer, E. S. & Stevanoviȍ, V. Rapid Prediction of Anisotropic Lattice Thermal Conductivity: Application to Layered Materials. *Chem. Mater.* **31**, 2048-2057 (2019).

29 Jiang, P., Qian, X., Yang, R. & Lindsay, L. Anisotropic thermal transport in bulk hexagonal boron nitride. *Phys. Rev. Mater.* **2**, 064005 (2018).

30 Jiang, P., Qian, X., Gu, X. & Yang, R. Probing Anisotropic Thermal Conductivity of Transition Metal Dichalcogenides MX_2 (M = Mo, W and X = S, Se) using Time-Domain Thermoreflectance. *Adv. Mater.* **29**, 1701068 (2017).

31 Jang, H., Wood, J. D., Ryder, C. R., Hersam, M. C. & Cahill, D. G. Anisotropic Thermal Conductivity of Exfoliated Black Phosphorus. *Adv. Mater.* **27**, 8017-8022 (2015).

32 Hsin, C.-L., Huang, J.-H., Spiewak, P., Ciupiński, Ł. & Lee, S.-W. Anisotropy of thermal conductivity in In_2Se_3 nanostructures. *Appl. Surf. Sci.* **494**, 867-870 (2019).

33 Ainsworth, L. Single Crystal Bismuth Telluride. *Proc. Phys. Soc. B* **69**, 606-612 (1956).

34 Li, C., Ma, H., Li, T., Dai, J., Rasel, M. A. J., Mattoni, A., Alatas, A., Thomas, M. G., Rouse, Z. W., Shragai, A., Baker, S. P., Ramshaw, B. J., Feser, J. P., Mitzi, D. B. & Tian, Z. Remarkably Weak Anisotropy in Thermal Conductivity of Two-Dimensional Hybrid Perovskite Butylammonium Lead Iodide Crystals. *Nano Lett.* **21**, 3708-3714 (2021).

35 Yu, Y., Cagnoni, M., Cojocaru-Mirédin, O. & Wuttig, M. Chalcogenide Thermoelectrics Empowered by an Unconventional Bonding Mechanism. *Adv. Funct. Mater.* **30**, 1904862 (2020).

36 Albanesi, E. A., Okoye, C. M. I., Rodriguez, C. O., Peltzer y Blanca, E. L. & Petukhov, A. G. Electronic structure, structural properties, and dielectric functions of IV-VI semiconductors: PbSe and PbTe. *Phys. Rev. B* **61**, 16589-16595 (2000).

37 Shakil, M., Akram, A., Zeba, I., Ahmad, R., Gillani, S. S. A. & Gadhi, M. A. Effect of mixed halide contents on structural, electronic, optical and elastic properties of $CsSnI_{3-x}Br_x$ for solar cell applications: first-principles study. *Mater. Res. Express* **7**, 025513 (2020).

38 Acharyya, P., Ghosh, T., Pal, K., Kundu, K., Singh Rana, K., Pandey, J., Soni, A., Waghmare, U. V. & Biswas, K. Intrinsically Ultralow Thermal Conductivity in Ruddlesden–Popper 2D Perovskite $Cs_2PbI_2Cl_2$: Localized Anharmonic Vibrations and Dynamic Octahedral Distortions. *J. Am. Chem. Soc.* **142**, 15595-15603 (2020).

39 Pailhès, S., Euchner, H., Giordano, V. M., Debord, R., Assy, A., Gomès, S., Bosak, A., Machon, D., Paschen, S. & de Boissieu, M. Localization of Propagative Phonons in a Perfectly Crystalline Solid. *Phys. Rev. Lett.* **113**, 025506 (2014).

40 Yang, J., Qian, X., Pan, W., Yang, R., Li, Z., Han, Y., Zhao, M., Huang, M. & Wan, C. Diffused Lattice Vibration and Ultralow Thermal Conductivity in the Binary Ln–Nb–O Oxide System. *Adv. Mater.* **31**, 1808222 (2019).

41 Seyf, H. R., Yates, L., Bougher, T. L., Graham, S., Cola, B. A., Detchprohm, T., Ji, M.-H., Kim, J., Dupuis, R., Lv, W. & Henry, A. Rethinking phonons: The issue of disorder. *NPJ Comput. Mater.* **3**, 49 (2017).

42 Seyf, H. R. & Henry, A. A method for distinguishing between propagons, diffusions, and locons. *J. Appl. Phys.* **120**, 025101 (2016).

43 Setyawan, W. & Curtarolo, S. High-throughput electronic band structure calculations: Challenges and tools. *Comput. Mater. Sci.* **49**, 299-312 (2010).
